# Cerium Oxide Nanoparticles Protect against Oxidant Injury and Interfere with Oxidative Mediated Kinase Signaling in Human-Derived Hepatocytes

**DOI:** 10.3390/ijms20235959

**Published:** 2019-11-27

**Authors:** Silvia Carvajal, Meritxell Perramón, Gregori Casals, Denise Oró, Jordi Ribera, Manuel Morales-Ruiz, Eudald Casals, Pedro Casado, Pedro Melgar-Lesmes, Guillermo Fernández-Varo, Pedro Cutillas, Victor Puntes, Wladimiro Jiménez

**Affiliations:** 1 Biochemistry and Molecular Genetics Service, Hospital Clínic Universitari, IDIBAPS, CIBERehd, 08036 Barcelona, Spain; silviacr_87@hotmail.com (S.C.); mperramon@clinic.cat (M.P.); dnise_2@hotmail.com (D.O.); jordi.ribera@ciberehd.org (J.R.); morales@clinic.cat (M.M.-R.); pmelgar@clinic.cat (P.M.-L.); guillermo.fernandez@ciberehd.org (G.F.-V.); wjimenez@clinic.cat (W.J.); 2 Department of Biomedicine, University of Barcelona, 08036 Barcelona, Spain; 3 School of Biotechnology and Health Sciences, Wuyi University, Jiangmen 529020, China; eudaldcm@gmail.com; 4 Cell Signalling and Proteomics Group, Centre for Haemato-Oncology, Barts Cancer Institute, Queen Mary University of London, London EC1M 6BQ, UK; p.m.casado-izquierdo@qmul.ac.uk (P.C.); p.cutillas@qmul.ac.uk (P.C.); 5 Institut Català de Recerca i Estudis Avançats, (ICREA), 08010 Barcelona, Spain; victor.puntes@icn.cat; 6 Vall d’Hebron Insitute of Research (VHIR), 08035 Barcelona, Spain; 7 Institut Català de Nanociència i Nanotecnologia (ICN2), 08193 Bellaterra, Spain

**Keywords:** cerium oxide nanoparticles, oxidative stress, human hepatic cells, phosphoproteomics, NAFLD

## Abstract

Cerium oxide nanoparticles (CeO_2_NPs) possess powerful antioxidant properties, thus emerging as a potential therapeutic tool in non-alcoholic fatty liver disease (NAFLD) progression, which is characterized by a high presence of reactive oxygen species (ROS). The aim of this study was to elucidate whether CeO_2_NPs can prevent or attenuate oxidant injury in the hepatic human cell line HepG2 and to investigate the mechanisms involved in this phenomenon. The effect of CeO_2_NPs on cell viability and ROS scavenging was determined, the differential expression of pro-inflammatory and oxidative stress-related genes was analyzed, and a proteomic analysis was performed to assess the impact of CeO_2_NPs on cell phosphorylation in human hepatic cells under oxidative stress conditions. CeO_2_NPs did not modify HepG2 cell viability in basal conditions but reduced H_2_O_2_- and lipopolysaccharide (LPS)-induced cell death and prevented H_2_O_2_-induced overexpression of MPO, PTGS1 and iNOS. Phosphoproteomic analysis showed that CeO_2_NPs reverted the H_2_O_2_-mediated increase in the phosphorylation of peptides related to cellular proliferation, stress response, and gene transcription regulation, and interfered with H_2_O_2_ effects on mTOR, MAPK/ERK, CK2A1 and PKACA signaling pathways. In conclusion, CeO_2_NPs protect HepG2 cells from cell-induced oxidative damage, reducing ROS generation and inflammatory gene expression as well as regulation of kinase-driven cell survival pathways.

## 1. Introduction

During the last few years, it has been suggested that antioxidants such as superoxide dismutase (Mn-SOD), resveratrol, colchicine, eugenol or vitamin E and C exert beneficial effects in chronic liver disease [1,2,3,4,5]. This hypothesis was raised based on the concept that the root of many hepatic disturbances involves an imbalance in reactive oxygen species (ROS) metabolism. Accordingly, any maneuver towards reversing this imbalance should improve liver functionality and diminish injury. In this regard, the therapeutic effects induced by antioxidants (vitamin C and SOD) on portal hypertension have already been shown in patients with liver disease [6] and CCl_4_-treated rats with portal hypertension [1]. Currently, the European and American guidelines for the management of patients with non-alcoholic fatty liver disease (NAFLD) support the use of vitamin E in nondiabetic adults with biopsy-proven NASH [7,8].

More recently a new player, namely cerium oxide nanoparticles (CeO_2_NPs), was incorporated to the group of antioxidant substances with therapeutic properties in experimental liver disease. CeO_2_NPs are a rare element belonging to the lanthanide series, with both Ce^3+^ and Ce^4+^ oxidation states that result in an autoregenerative redox cycle between the two states, followed by the capture or release of oxygen [9]. As a consequence, the oxygen storage capacity of CeO_2_NPs becomes very useful to eliminate free radicals while generated in situations of ROS imbalance [10,11]. Recently, we have demonstrated that CeO_2_NPs reduce steatosis, portal hypertension and display anti-inflammatory properties in rats with experimental liver fibrosis [12]. Also, we observed an antilipogenic and anti-inflammatory effect in the liver of rats subjected to a methionine and choline deficient diet for six weeks [13]. A major difference between antioxidants such as SOD or vitamin C and CeO_2_NPs is that the former two are rapidly oxidized or degraded (metabolized) whereas CeO_2_NPs act as self-renewal catalysts. In addition, CeO_2_NPs only display biological effects in the case of ROS overproduction; otherwise, they behave as inert inorganic material [14]. For this reason, CeO_2_NPs are expected to behave as permanent vitamin C or SOD-like effectors and perform better than natural antioxidants, being specifically active in the case of inflammation. This makes CeO_2_NPs especially helpful to arrest NAFLD progression, which is critically dependent on chronic effects of ROS in the liver. It is therefore important to understand the mechanisms of action of CeO_2_NPs to ensure safe use in human liver disease.

In this investigation, we address this issue by assessing whether CeO_2_NPs are able to prevent or attenuate the oxidant-mediated injury induced by H_2_O_2_ or lipopolysaccharide (LPS) in HepG2 cells, which is a cell line derived from human hepatocytes that preserve most of the morphological and metabolic characteristics of the original hepatic cells [15,16]. The aim of the study was to further elucidate whether the therapeutic effect of CeO_2_NPs already observed in experimental liver disease can be translated to human cells and to investigate the molecular mechanisms involved in this beneficial effect.

## 2. Results

### 2.1. Cerium Oxide Nanoparticles Characterization

**Figure 1:**
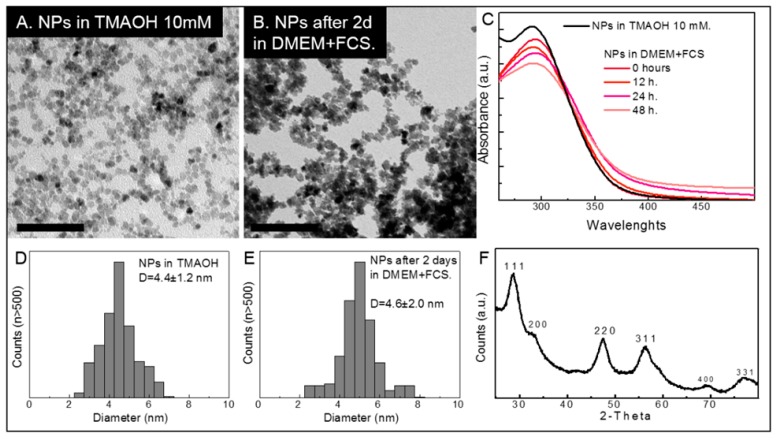
Characterization of the CeO_2_NPs used in this work. (**A**) NPs after purification and resuspension in Tetramethylammonium hydroxide (TMAOH) 10 mM. Scale bar is 50 nm. (**B**) NPs after 2 days in Cell Culture Medium (CCM), Dulbecco’s Modified Eagle Medium (DMEM) + 10% Fetal Calf Serum (FCS). Scale bar is 50 nm. Although TEM images of the NPs in the CCM are not as clear as in TMAOH, due to the presence of other components of the media, NPs can still be observed and measured. (**C**) Evolution of the UV-VIS spectra of CeO_2_NPs in the CCM. NPs are stable throughout the time of the experiments and presence of large agglomerates can be ruled out since those agglomerates would increase the absorbance at larger wavelengths. (**D**) and (**E**) Size distribution using ImageJ free software (Available online: https://imagej.nih.gov/ij/) counting more than 500 NPs of different TEM images of the NPs in TMAOH and in CCM. A slight increase can be observed in the size distribution (standard deviation increases from 1.2 to 2.0) probably due to dissolution of NPs in the more aggressive media of the CCM. However, after 2 days, mean size is unaltered. (**F**) X-ray Diffraction (XRD) spectra of the CeO_2_NPs in TMAOH 10 mM showing the characteristic peaks of CeO_2_ crystals.

The CeO_2_NPs used in this work and their evolution in the physiological media have been already described extensively in our previous works [12,17], and the results presented here are similar to the ones already presented, i.e., the nanoparticles (NPs) are stable during their time in the physiological media and a protein corona is made on their surface during that time. This can be observed in Figure 1 and Table 1 as size distribution from Transmission Electron Microscopy (TEM) images is only slightly modified, Z-potential decreases to the value of the fetal calf serum, while the hydrodynamic diameter measured by Dynamic Light Scattering (DLS) increases and Ultraviolet-Visible (UV-VIS) spectra show some modifications (both from the slight modification of the size distribution and the absorption of proteins).

### 2.2. CeO_2_NPs Protect HepG2 Cells from H_2_O_2_- and LPS-induced Cytotoxicity

**Figure 2:**
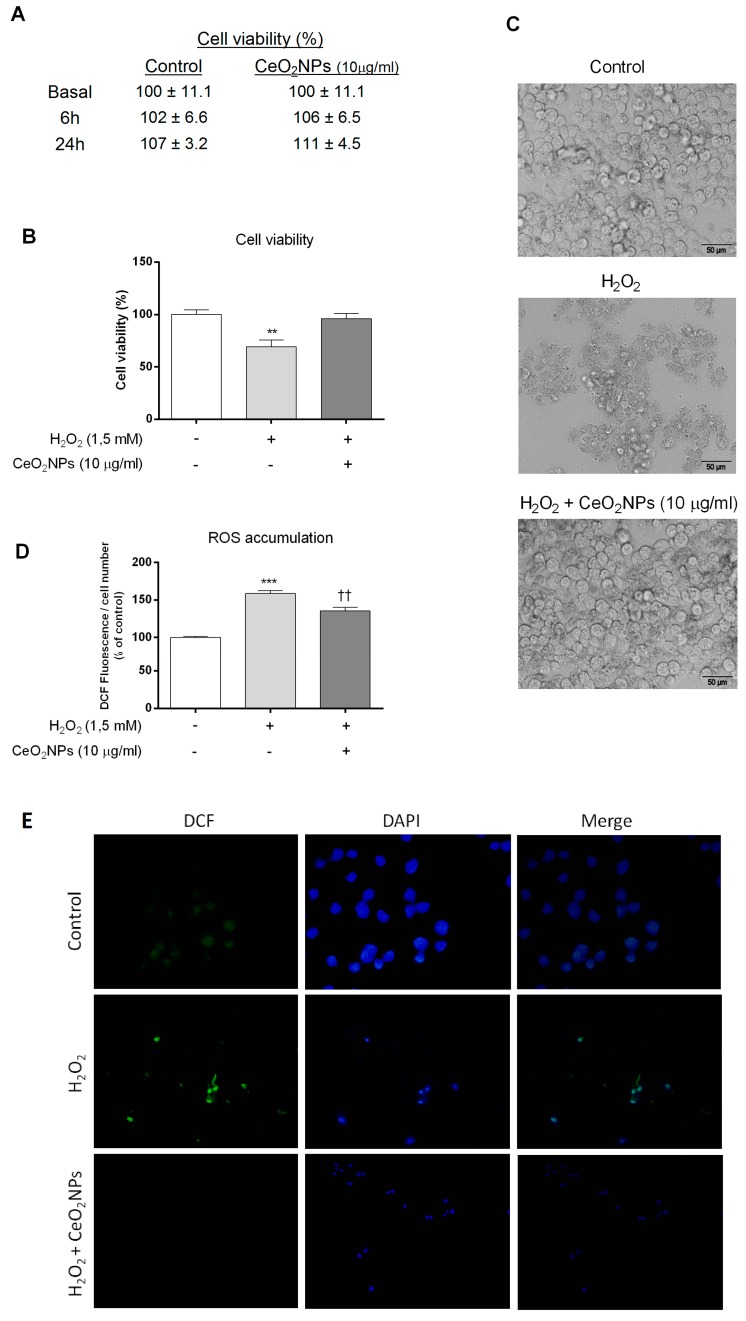
CeO_2_NPs inhibited H_2_O_2_-induced cytotoxicity in HepG2 cells. (**A**) Viability of HepG2 cells after treatment with CeO_2_NPs (10 µg/mL) determined using the MTS assay (MTS) at indicated time points. Quadruplicates of each group were used in each independent experiment. The results are expressed as percentage of control cells for the times indicated. (**B**) HepG2 cells were exposed to 1.5 mM H_2_O_2_ and treated with 10 µg/mL of CeO_2_NPs for 1.5 h. Cell viability was detected using MTS and expressed as percentage of control cells. Data are the mean ± S.E. of triplicate experiments. ** *p* < 0.01 vs. control. + presence; − absence. (**C**) Representative phase contrast light microscopy images of HepG2 cells at 1.5 h after H_2_O_2_ treatment. (**D**) Reactive oxygen species (ROS) production was determined by fluorescence spectrophotometry using the oxidant-sensitive dye 2’,7’-DCF-HDA. The results were expressed as percentage of control cells for the treatments indicated. *** *p* < 0.001 vs. control; †† *p* < 0.01 vs. H_2_O_2_. + presence; − absence. (**E**) Representative microphotographs of DFC fluorescence (DCF, green) and 4’,6-diamidino-2-phenylindole (DAPI, blue) after H_2_O_2_ treatment (original magnification, 200×).

**Figure 3:**
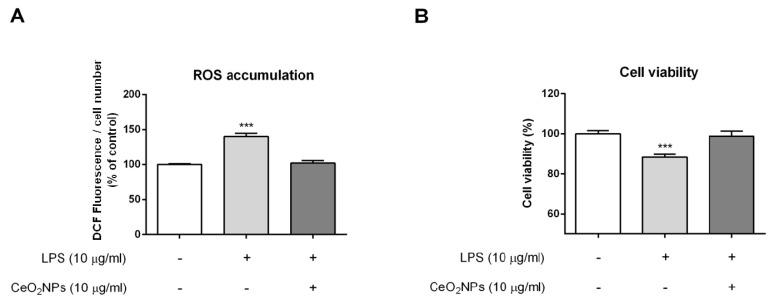
CeO_2_NPs reduced lipopolysaccharide (LPS)-induced ROS production and cytotoxicity in HepG2 cells. (**A**) Cells were treated with 10 µg/mL LPS for 2 h in the presence of CeO_2_NPs (10 µg/mL) or vehicle. Extracellular ROS production was determined by fluorescence spectrophotometry using the oxidant-sensitive dye 2’,7’-DCF-DA. The results were expressed as percentage of control cells for the indicated treatments. Data are mean ± S.E. *** *p* < 0.001. (**B**) HepG2 cells were exposed to 10 µg/mL LPS and treated with 10 µg/mL CeO_2_NPs or vehicle for 24 h. Cell viability was detected using MTS and expressed as percentage of control cells. Data are the mean ± S.E, *** *p* < 0.001; + presence; − absence.

To demonstrate that the CeO_2_NPs employed are non-toxic to HepG2, cells were exposed to CeO_2_NPs, and cell survival was assessed using the MTS assay. As shown in Figure 2A, we did not find significant differences in cell death between control and CeO_2_NPs-exposed cells up to concentrations of 10 µg/mL of CeO_2_NPs. Next, we further certified the ability of CeO_2_NPs to shield HepG2 cells from oxidative damage. Cells were incubated with H_2_O_2_ or LPS to promote oxidative stress and were then treated with different doses of CeO_2_NPs. Figure 2B,D,E show the effect of CeO_2_NPs on cell viability and ROS production, respectively. As anticipated, the presence of H_2_O_2_ significantly increased the oxidation of dichlorofluorescin diacetate (DCF-DA) in HepG2 cells, in association with a reduction in cell viability. Remarkably, both effects were reverted when HepG2 cells were coincubated with 10 µg/mL of CeO_2_NPs. Similar results were obtained on measuring glutathione (GSH) concentration. H_2_O_2_ treatment reduced GSH concentrations to 37% ± 5% from the basal value, whereas subsequent administration of CeO_2_NPs recovered these figures to 64% ± 5% (*p* < 0.01). Furthermore, cellular morphological visualization using light microscopy (Figure 2C) showed that most of the HepG2 cells lost their normal morphology when stimulated with H_2_O_2_, whereas this change was absent when cells were treated with CeO_2_NPs. A similar pattern of response was found when cells were stimulated with LPS. Indeed, LPS increased ROS production and decreased cell viability, and CeO_2_NPs prevented these effects in HepG2 cells (Figure 3). These results indicate that CeO_2_NPs treatment reduces ROS accumulation and the associated cell death induced by H_2_O_2_ and LPS in HepG2 cells.

### 2.3. Expression Profile of Genes Related to Oxidative Stress in HepG2 Cells Exposed to H_2_O_2_

The relative expression of 84 genes associated with oxidative stress and antioxidant protection in HepG2 cells exposed to H_2_O_2_ and treated with CeO_2_NPs was assessed using a commercially available PCR array. Table 2 depicts the 25 out of the 84 investigated genes showing a 2-fold or superior change in expression between H_2_O_2_-exposed and control cells and the expression of genes affected by CeO_2_NPs treatment compared to non-treated H_2_O_2_-exposed cells. Nine genes were significantly upregulated in H_2_O_2_-exposed cells. This group included genes encoding peroxidase and reductase enzymes involved in antioxidant metabolism (MPO, PTGS1, TXNRD1 and SRXN1), genes related to ROS metabolism (NCF1), as well as oxidative stress responsive genes, namely DUSP1, GCLC, GCLM, and HSPA1A. On the other hand, 12 genes appeared to be significantly downregulated, including genes encoding antioxidant enzymes (CAT, GPX7 and SOD3), genes controlling ROS production (UCP2 and EPHX2), oxidative stress responsive genes (DCHR24, FOXM1, MBL2, OXR1, SCARA3, and SEPP1), and the oxygen transporter MB.

**Figure 4:**
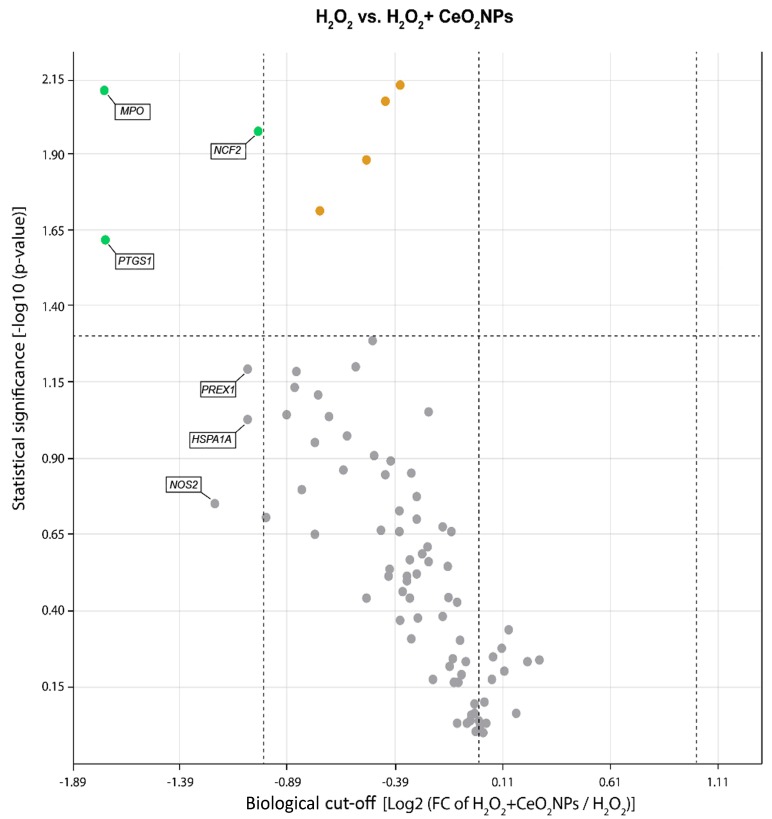
A volcano plot representation of the differentially expressed genes in a pair-wise comparison of untreated and CeO_2_NP-treated H_2_O_2_-exposed HepG2 cells. Significance was set to a *p* value based on a Student’s *t*-test of 0.05 [−log10 (*p*-value) ≥ 1.30], the biological cut-off was set to a fold regulation of ± 2 fold [−1 ≥ log2 (FC of H_2_O_2_+CeO_2_NPs/H_2_O_2_) ≥ 1]. In accordance with these two criteria, the top seven deferentially expressed genes are labeled with their corresponding gene ID. The different color codes used represent insignificant genes (grey), both biologically and statistically significant down-regulated genes (green) and statistically but not biologically significant down-regulated genes (orange) in CeO_2_NP treated cells.

**Figure 5:**
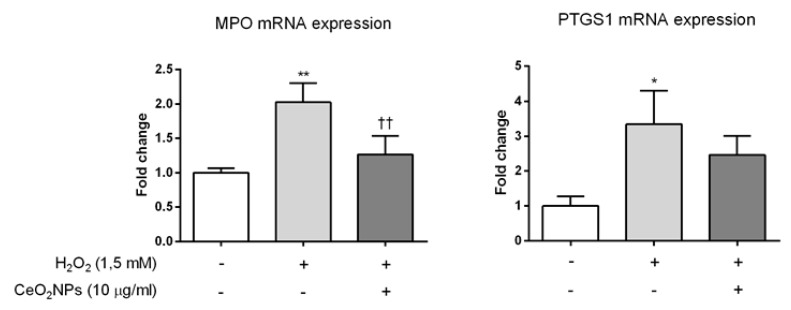
Effect of CeO_2_NPs on the expression of oxidative stress-related genes in HepG2 cells exposed to H_2_O_2_. Cells were stimulated with 1.5 mM H_2_O_2_ for 24 h and incubated in the absence or presence of CeO_2_NPs (10 µg/mL). The messenger RNA expression of MPO and PTGS1 was assessed using real-time PCR. Data are the mean + S.E. of triplicate experiments. * *p* < 0.05; ** *p* < 0.01 vs. control; †† *p* < 0.01 vs. H_2_O_2_.

A 2-fold or greater change in expression with *p* < 0.05 was considered statistically significant on comparing the untreated vs. the CeO_2_NPs treated H_2_O_2_-exposed cell groups. A volcano plot of the data is presented in Figure 4. CeO_2_NPs significantly decreased the expression of two genes with peroxidase activity (MPO, PTGS1) and a gene encoding a subunit of NADPH oxidase (NCF2). These changes in mRNA expression induced by CeO_2_NPs in H_2_O_2_-treated cells were confirmed on assessing messenger abundance using real time-PCR (Figure 5).

### 2.4. CeO_2_NPs Reduce H_2_O_2_-Induced Expression of iNOS in HepG2 cells

**Figure 6:**
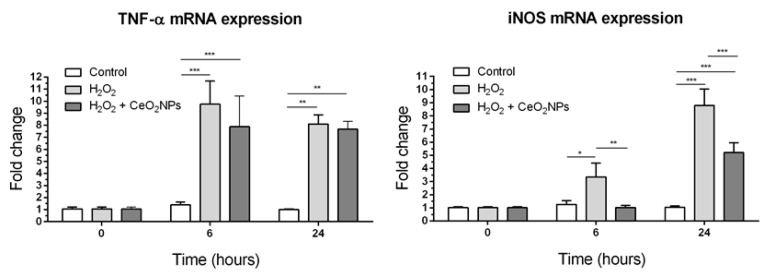
Effect of CeO_2_NPs on the expression of pro-inflammatory genes in HepG2 cells exposed to H_2_O_2._ Cells were stimulated with 1.5 mM H_2_O_2_ for 6 and 24 h and incubated in the absence or presence of CeO_2_NPs (10 µg/mL). The messenger RNA expression of iNOS and TNF-α was then assessed using real-time PCR. Data are the mean ± S.E. of triplicate experiments. * *p* < 0.05; ** *p* < 0.01; *** *p* < 0.001.

Since CeO_2_NPs are able to reduce H_2_O_2_-induced ROS production in HepG2 cells and also modify the expression of oxidative stress-related genes, we investigated whether the expression of key pro-inflammatory genes (TNF-α and iNOS) could be abrogated by CeO_2_NPs. As anticipated, H_2_O_2_ exposure increased mRNA expression of TNF-α and iNOS in HepG2 cells. Moreover, CeO_2_NPs exerted a specific inhibitory effect on iNOS expression since exposure of H_2_O_2_-treated cells to CeO_2_NPs markedly reduced iNOS but not TNF-α expression in these cells (Figure 6).

### 2.5. Identification of Signaling Networks Affected by Oxidative Stress in HepG2. Effect of CeO_2_NPs

**Figure 7:**
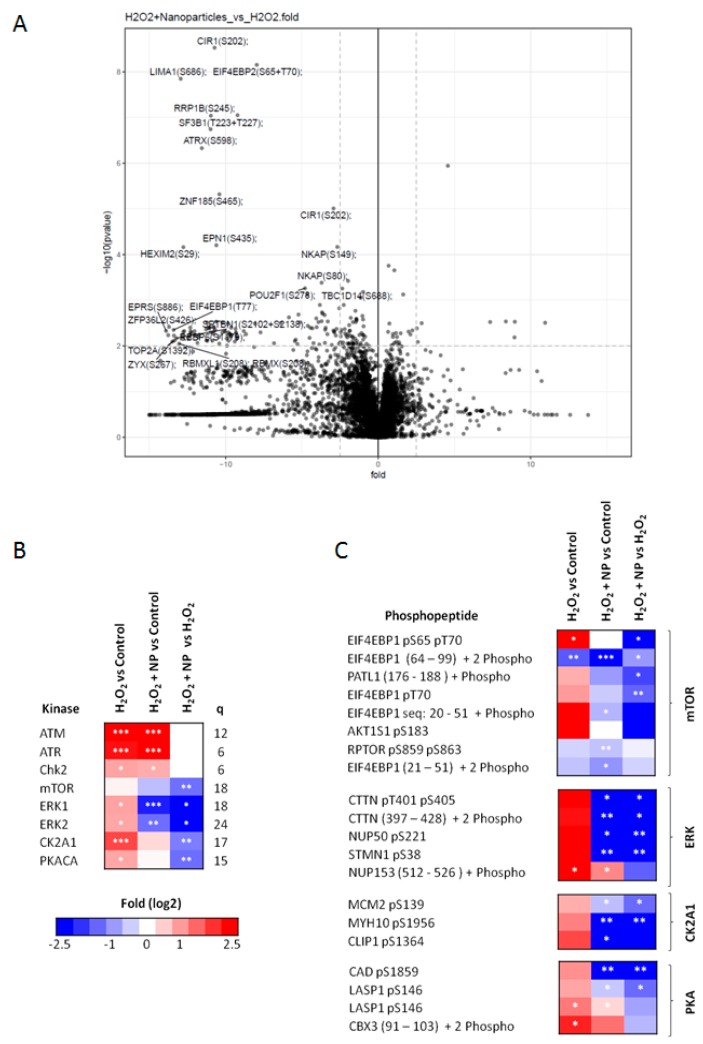
Effect of CeO_2_NPs on the modulation of kinase activity in HepG2 cells exposed to H_2_O_2_. (**A**) Volcano plot showing the fold difference between H_2_O_2_-exposed HepG2 cells treated or not with CeO_2_NPs for 1 h. Fold difference in peptide abundance is represented as the log2 (a positive value for the log2 of the fold difference indicates the increased abundance of a phosphorylated peptide after CeO_2_NPs treatment) and *p* value as −log10 (a significance −log10 *p* value > 2 corresponds to a linear *p* value of < 0.01). (**B**) Heatmap showing the enrichment of substrate groups for the different kinases calculated by the KSEA algorithm with the PhosphoSite database and the z-score method of calculating enrichment. The extent of enrichment was calculated as the abundance of substrate phosphorylation peptide in HepG2 cells under one condition divided by its abundance in another condition. Specifically, the first column shows the result of KSEA for cells exposed to H_2_O_2_ vs. control cells treated with vehicle, the second column shows KSEA of cells exposed to H_2_O_2_ and CeO_2_NPs vs. control treated with vehicle, and the third column shows KSEA of cells exposed to H_2_O_2_ and CeO_2_NPs vs. HepG2 exposed to H_2_O_2_. Column “q” indicates the number of phosphopeptides used to estimate the enrichment in kinase activity for the indicate kinase. An arbitrary cut-off value of 6 phosphopeptides has been used. (**C**) Heatmap showing phosphopeptides significantly affected by exposure of HepG2 cells to H_2_O_2_ and H_2_O_2_ + CeO_2_NPs in proteins linked to mTOR, ERK, CK2A1 and PKA signaling pathways. Statistical significant was assessed using *t*-test in (**A**,**C**) or *z*-test (**B**). * *p* < 0.05; ** *p* < 0.01; *** *p* < 0.001.

To investigate the effects of CeO_2_NPs on kinase driven signaling pathways we used mass spectrometry phosphoproteomics. We identified and quantified a total of 10,210 phosphopeptides in four independent biological replicates. As anticipated, a large number were affected by H_2_O_2_ treatment. At arbitrary threshold values of ±0.8-fold change (log2) and a *p* value of < 0.05, data analysis revealed that the phosphorylation of 1503 peptides (1037 increased and 466 decreased) was affected after incubation of HepG2 cells with H_2_O_2_ (1.5 mM) for 60 min. Interestingly, the phosphorylation of a substantial number of peptides was affected by CeO_2_NPs treatment (Figure 7A). Following exposure to CeO_2_NPs, the intensity of phosphorylation went back toward normal values in 39 out of the 1037 peptides with increased phosphorylation; whereas, none of the 466 peptides in which H_2_O_2_ induced a reduction in the intensity of the phosphorylation partially or totally recovered following CeO_2_NPs incubation (Table 3). A number of phosphorylation sites were observed on proteins linked to cell proliferation, stress response, cytoskeletal signaling and gene transcription regulation. Interestingly and consistently with our gene expression data (Figure 4), TERF2 and ARID1A, which are two of the most frequently altered genes in hepatocellular carcinoma (HCC), were among these peptides (Table 3).

Previous investigations have demonstrated that kinase activity can be estimated by measuring the phosphorylation state of known substrates using kinase substrate enrichment analysis (KSEA) [18,19]. We used this computational approach to elucidate kinases involved in the effects of CeO_2_NPs. As expected, KSEA showed that H_2_O_2_ produced an increase in the estimated activity of ATM, ATR or CHK2, which are kinases involved in DNA damage response (Figure 7B). This analysis also indicated that CeO_2_NPs interfered with mTOR, MEK/ERK, CK2A1 and PKA signaling (Figure 7B). Thus, CeO_2_NPs reversed the ability of H_2_O_2_ to induce the phosphorylation of mTOR substrates like 4EBP1 and PRAS40 (also known as AKT1S1, Figure 7C). In addition, we observed reduced phosphorylation of proteins involved in the PI3K/mTOR pathway including ACIN1, PRKDC and YAP1 (Table 3). The nanoparticles also inhibit the phosphorylation of multiple ERK substrates including Cortactin, Stahmin and NP50. In addition, NPs block the phosphorylation induced by the H_2_O_2_ treatment on NP150, another ERK substrate (Figure 7C).

CK2A1 phosphorylates a wide array of substrates and regulates several cellular processes, including cell cycle progression and transcription. HepG2 exposure to CeO_2_NPs also resulted in dephosphorylation of several peptides known to be substrates of CK2A1, such as MCM2 at S139, MYH10 at S1956 and CLIP at S1364 (Figure 7C). In addition to the PhosphoSite annotated substrates, other sites linked to CK2A1 like kanadaptin at S466, MCM3 at S711 and T713, CHMP2B at S199, and RBMX at S208 also followed the same trend (Table 3). Following CeO_2_NPs treatment, we also observed dephosphorylation of POU2F1, a substrate of PKACA, a kinase that phosphorylates a wide variety of substrates in the cytoplasm and the nucleus and regulates trafficking of compartmentalized pools of its regulatory subunits (Figure 7C). In summary, an assessment of the phosphoproteome in cells exposed to oxidative stress indicates that CeO_2_NPs negatively interfere with the signaling of mTOR, ERK 1/2, CK2A1 and PKACA. Moreover, CeO_2_NPs also dephosphorylated TERF2 and ARID1A, which are major therapeutic targets in HCC.

## 3. Discussion

The recent description that nanoceria could be therapeutically useful in pathological conditions characterized by enhanced oxidative stress and inflammation [20], including liver disease, raised the possibility of using this material in patients. However, assessment of the beneficial effects of CeO_2_NPs in humans should be made with caution. As a first step to address this issue, the current study sought to investigate the effect of CeO_2_NPs in a human hepatic cell line when challenged with a well characterized pro-oxidant or proinflammatory agent such as H_2_O_2_ or LPS. Oxidative stress and inflammation are considered key mechanisms of progression to NASH, fibrosis and/or hepatocellular carcinoma in patients with NAFLD.

In the current investigation, exposure of human HepG2 cells to 10 µg/mL of CeO_2_NPs did not alter cell viability under normal conditions. This is coincident with previous investigations demonstrating that at the concentration used these nanoparticles are non-toxic to numerous mammalian cells, including endothelial, breast and fibrosarcoma cells [21,22]. This feature seems to be characteristic of CeO_2_NPs since other metal oxide nanoparticles, such as zinc oxide and titanium (exposed to UV-light), have displayed remarkable toxicity [23].

CeO_2_NPs have been proposed as a potential treatment for clinical conditions in which increased oxidative stress plays a significant pathogenic role. Actually, CeO_2_NPs display superoxide dismutase (SOD) and catalase mimetic activities [10,24,25] and present hydroxyl radical scavenging properties, thus resulting in a reduction of ROS [24,25]. This has been further supported by experimental evidence demonstrating the ROS-scavenging potential of CeO_2_NPs in medicine [26]. Oxidative stress plays a critical role in the development of chronic liver damage and stimulates its progression. It is well established that oxidative stress constitutes the background of viral, alcoholic liver diseases, non-alcoholic steatohepatitis and participates in the fibrogenic response of the liver [27]. Therefore, this investigation was addressed to assess whether CeO_2_NPs could protect hepatocyte injury induced by a direct prooxidant stimulus such as H_2_O_2_. Our results showed that CeO_2_NPs reduced the cellular cytotoxicity induced by H_2_O_2_, which was associated with a decrease in cellular oxidative stress. Next, we further investigated the ability of CeO_2_NPs to decrease the endogenous production of ROS by stimulating HepG2 cells with LPS. This bacterial wall-derived product is a well-known inducer of ROS production in several cell lines [28,29]. In line with these investigations, we showed that LPS treatment increased ROS production in HepG2 cells (Figure 3A). Furthermore, the presence of CeO_2_NPs blocked LPS-induced ROS production in these cells. This phenomenon occurred in the absence of significant modifications in cell viability. Altogether, these results show that CeO_2_NPs also protect HepG2 cells from LPS-induced oxidative stress.

Although the physicochemical properties of CeO_2_NPs as a ROS scavenger are well described, the mechanisms of their effects on biological systems remain largely unknown. In order to investigate the specific molecular mechanisms by which CeO_2_NPs exert the protective effects observed in HepG2 cells, we studied the pattern of expression of a wide array of genes involved in oxidative stress and antioxidant defense. As expected, treatment of HepG2 cells with H_2_O_2_ significantly altered the biological response to ROS with significant changes in the expression of 30% of the genes studied (25 of 84 genes). Interestingly, CeO_2_NPs treatment of H_2_O_2_-stimulated cells allowed the identification of five specific genes related to the cellular response of CeO_2_NPs to ROS. These include important oxidative genes such as myeloperoxidase (MPO), prostaglandin-endoperoxide synthase 1 (PTGS1), also known as cyclooxygenase 1 (COX1), neutrophil cytosolic factor 2 (NCF2, also known as P67PHOX), and inducible nitric oxide synthase (iNOS). The MPO enzyme catalyzes the conversion of hydrogen peroxide to hypochlorite and hypochlorous acid, and its activation has been related to the proapoptotic and profibrotic pathway of progression in non-alcoholic fatty liver disease [30,31]. The PTGS1 enzyme catalyzes the conversion of arachidonic acid to prostaglandins and thromboxane, and it is widely known that PTGS1 disruption translates into reduced inflammatory response [32,33]. NCF2 encodes a 67 kDa cytosolic subunit that is required for activation of NADPH oxidase to produce superoxide anions. iNOS is a major enzyme in the synthesis of nitric oxide. Overproduction of nitric oxide by iNOS is a critical mediator of inflammation contributing to tissue injury. It is interesting to note that iNOS transfers electrons from NADPH in the NO synthesis reaction and that iNOS expression requires NADPH oxidase-dependent redox signaling [34,35]. In addition, iNOS can also catalyze the production of superoxide ion. In our experiments, the 5.3- 3.7- and 8.7-fold induction of MPO, PTGS1, and iNOS genes, respectively, caused by H_2_O_2_ was almost normalized when HepG2 cells were treated with CeO_2_NPs. Furthermore, a significant reduction in NCF2 expression was also detected in H_2_O_2_-exposed cells when treated with CeO_2_NPs. Our results therefore suggest that, besides the inherent antioxidant properties of the CeO_2_NPs chemistry, the cytoprotective effects induced by CeO_2_NPs are mediated by the reduction in the expression of these oxidative enzymes.

It is noteworthy that increased expression of these genes was also found in the liver of rats with CCl_4_-induced fibrosis [12]. Moreover, treatment with CeO_2_NPs significantly reduced the hepatic expression of iNOS and NCF2 in this model of liver disease [12]. The similarity of the biological effects observed after CeO_2_NPs administration in the injured liver of experimental animals and that observed in the current study in HepG2 cells cultured under pro-oxidant conditions further support the potential therapeutic usefulness of CeO_2_NPs in human liver disease.

The impact of CeO_2_NPs on cell phosphorylation in human hepatic cells under oxidative stress conditions has not been systematically investigated using untargeted MS-based proteomics. Therefore, we studied the effect of CeO_2_NPs in an oxidative stress model that involves exposing cells to H_2_O_2_ (1.5 mM). In other words, we investigated how CeO_2_NPs affect the H_2_O_2_-induced phosphoproteome changes in human-derived hepatocytes. We found that 10% of all the peptides assessed were phosphorylated as a result of exposure to H_2_O_2_. However, the effect of CeO_2_NPs was considerably more selective. Actually, a reduction in phosphorylation was significantly observed in 39 out of the 1037 peptides affected by H_2_O_2_ exposure. A significant number of proteins dephosphorylated as a result of exposure to CeO_2_NPs were linked to cell proliferation and gene transcription, including ACIN1, YAP1 and 4E-BP1. The activities of kinases are linked to the wiring of signaling networks [36], thus several tools have been developed to link phosphorylation data to upstream kinases based on phosphorylation motifs [37,38]. In the current investigation we have inferred kinase pathway activation based on values of substrate group enrichment obtained from previous knowledge of the kinase-substrate relationship. As a result, we found that H_2_O_2_-exposed cells treated with CeO_2_NPs had decreased amounts of phosphorylated substrates of mTOR, ERK 1/2 and PKACA, all of which have known roles in promoting cell growth, angiogenesis and carcinogenesis. Moreover, CeO_2_NPs also dephosphorylated TERF2 and ARID1A, which play crucial roles in the initiation and development of HCC [39,40,41,42].

As a limitation of the study, the toxicity of CeO_2_NPS was not evaluated in a normal hepatic cell line. However, Gaiser et al. reported no toxicity of CeO_2_NPs in the C3A human hepatocyte cell line at doses up to 100 times higher than those used in our study [43]. More recently, Singh et al. also did not find toxicity of CeO_2_NPs in the human hepatic cell line WRL-68 [44]. In addition, although no in vivo or functional experiments were performed to verify the efficacy of CeO_2_NPs in reducing oxidative damage and inflammatory response, recent results in animal models of liver disease have consistently shown antioxidant and anti-inflammatory effects of CeO_2_NPs in the liver [12,13,45]. Finally, further studies are necessary to confirm that the specific mechanism of oxidative damage reduction identified in this study can be translated to primary human hepatic cells and in in vivo conditions.

## 4. Material and Methods

### 4.1. Synthesis and Characterization of CeO_2_NPs

CeO_2_NPs were synthesized by the chemical precipitation of cerium (III) nitrate hexahydrated (Sigma-Aldrich, St. Louis, MO, USA) in a basic aqueous solution [17]. By modifying the pH conditions, different sizes can be obtained. Here, we used 4 nm NPs at a concentration of 1 mg/mL. In a first step, 10 mM of cerium (III) nitrate hexahydrate was dissolved in 100 mL of absolute ethanol at room temperature. The solution was left under stirring for about 30 min. One mL of TMAOH (1.0 ± 0.02 M in H_2_O) was added to the 100 mL solution at a final concentration of 10 mM, and the mixture was left under stirring. NPs were purified using centrifugation and resuspended in aqueous solution of 10 mM TMAOH, which acts as a stabilizer. The surface charge of the NPs was characterized in a Z-sizer (Malvern, Worcestershire, UK), while the crystal size was characterized using high-resolution TEM (HR-TEM) in a Tecnai G2 F20 at 200 kV (FEI, Hillsboro, Oregon, USA) and XRD (Xpert Pannalytical, Westborough, MA, USA), and the light interaction was characterized using UV-VIS spectroscopy (Shimadzu, Kyoto, Japan). Size distribution was computer analyzed by ImageJ (National Institutes of Health, Bethesda, MD, USA). CeO_2_NPs were kept at 4 °C until used. CeO_2_NPs were then diluted with DMEM (Life technologies, Carlsbad, CA, USA) to the final concentration 0.1, 1 or 10 μg/mL.

### 4.2. Cell Culture and Treatment

All studies were conducted with HepG2 human hepatocytes derived from a liver hepatocellular carcinoma obtained from the American Type Culture Collection (ATCC Cat# HB-8065, RRID: CVCL_0027; Manassas, VA, USA). This immortalized, stable cell line can be repeatedly frozen, thawed and propagated. Cells were grown to confluence for 24 h in DMEM, supplemented with 50 U/mL penicillin, 50 µg/mL streptomycin and 10% FCS, in a humidified atmosphere in 5% CO_2_ at 37 °C. Thereafter, cells were switched to serum-free medium for 24 h. For cell stimulation and treatment, the old medium was removed and replaced with medium containing 1.5 mM H_2_O_2_ (Sigma-Aldrich, St. Louis, MO, USA) and CeO_2_NPs (10 µg/mL) or vehicle (TMAOH, 0.17 mM), respectively. In preliminary experiments we observed that concentrations of CeO_2_NPs higher than 20 µg/mL may result in a significant diminution of cell viability. On the other hand, the concentration of H_2_O_2_ was selected based on preliminary experiments showing that 60 min after H_2_O_2_ loading the 1.5 mM dose resulted in a maximal effect on DCFH concentration without affecting cell survival. Higher concentrations of H_2_O_2_, however, resulted in a dramatic decrease in survival. Cells were incubated for the indicated time points and then harvested for biochemical or molecular assays. All experiments were repeated at least three times.

Cell viability analysis: Cell viability was determined using MTS methodology (CellTiter 96; Promega, Madison, WI, USA) according to the manufacturer’s instructions. In brief, cells were seeded (1 × 10^5^ cells/well) in 96-well plates and treated with H_2_O_2_ or LPS (10 µg/mL) and CeO_2_NPs as described above. Cells were washed with Hank’s Balanced Salt Solution (HBSS), and CellTiter reagent was added to each well. After incubation for 3 h at 37 °C to allow cells to bioreduce MTS into formazan, the absorbance of the formazan was measured with a spectrophotometer (FLUOstar OPTIMA; BMG LABTECH, Ortenberg, Germany) at 492 nm. The quantity of formazan is directly proportional to the number of living cells in culture.

ROS measurement: Fluorescence spectrophotometry was used to measure ROS, with 2’,7’-DCF-DA as the probe (Master Probes, Invitrogen Labs, Thermo Fisher Scientific, Waltham, MA, USA). DCF-DA readily diffuses through the membrane and is enzymatically hydrolyzed by intracellular esterases to the nonfluorescent DCFH, which can then be rapidly oxidized to fluorescent DCF in the presence of ROS. Cells incubated alone or treated with H_2_O_2_ or LPS in the presence of CeO_2_NPs or vehicle were washed with HBSS and incubated with 10 μM DCF-DA in DMEM for 40 min at 37 °C in the dark. The cells were trypsinized and diluted followed by staining with 0.02% trypan blue. The number of cells stained with trypan blue was counted under a light microscope. The supernatant was collected to measure ROS production, and the intensity of fluorescence was immediately read in a fluorescence spectrophotometer (FLUOstar OPTIMA; BMG LABTECH, Ortenberg, Germany) at 485 nm for excitation and at 520 nm for emission. Additionally, in these experiments we also measured GSH concentration. GSH was determined using the Abcam Inc. detection assay kit (ab138881) (Abcam, Cambridge, UK) according to the manufacturer’s guidelines. The samples were prepared by lysis of total cell proteins in PBS/0.5% NP-40 lysis buffer followed by a 1:50 dilution. Serial dilutions of GSH standards were prepared along with assay mixtures for the detection of GSH using 100× Thiol green solution, incubated for 30 min and read at 490/520 nm.

mRNA expression of inflammatory genes in cultured cells: HepG2 cells were seeded (8 × 10^5^ cells per well) in 12-well plates and incubated alone or treated with H_2_O_2_ in the presence of CeO_2_NPs (10 μg/mL) or vehicle for 6 and 24 h. Total RNA from cultured cells was extracted using the commercially available kit: TRIZOL (TRI Reagent; Sigma-Aldrich, St. Louis, MO, USA). The RNA concentration was determined using spectrophotometric analysis (ND-100 spectrophotometer; Thermo Fisher Scientific, Waltham, MA, USA). One microgram of total RNA was reverse-transcribed using a cDNA synthesis kit (High-Capacity cDNA Reverse Transcription Kit; Applied Biosystems, Foster City, CA, USA). Primers and probes for human TNFα (left: 5’-CGCTCCCCAAGAAGACAG-3’, right: 5’-CTGCCACGATCAGGAAGG-3’; probe number 73), inducible nitric oxide synthase (iNOS) (left: 5’-TGCATGGATAAGTACAGGCTGA-3, right: 5’-CCATTGCCAAACGTACTGGT-3’; probe number 66), myeloperoxidase (MPO) (left: 5’-CGTCAACTGCGAGACCAG-3’, right: 5’-GTCATTGGGCGGGATCTT-3’; probe number 66), prostaglandin-endoperoxide synthase 1 (PTGS1) (left: 5’-TTCTCTCGCCAGATTGCTG-3’, right: 5’-CCGAGACTCCCTGATGACA-3’; probe number 76) and hypoxanthine-guanine phosphoribosyltransferase (HPRT) used as an endogenous standard (left: 5’-TGACCTTGATTTATTTTGCATACC-3’, right: 5’-CGAGCAAGACGTTCAGTCCT-3’; probe number 73) were designed according to human TNFα, iNOS, MPO, PTGS1 and HPRT sequences (GenBank NM_000594.2, NM_000625.4, NM_000250.1, NM_16931.3, NM_000962.2 and NM_000194.2, respectively) to include intron spanning using the Universal ProbeLibrary Assay Design Center through ProbeFinder version 2.5 software (Roche Diagnostics, Indianapolis, IN, USA; Available online: http://lifescience.roche.com/shop/en/mx/overviews/brand/universal-probe-library). Real-time quantitative polymerase chain reaction was analyzed in duplicate and performed with the LightCycler 480 (Roche Diagnostics, Basel, Switzerland), as previously described [46,47]. A 10-µL total volume reaction of diluted 1:8 cDNA, 200 nM primer dilution, 100 nM prevalidated 9-mer probe (Universal ProbeLibrary) and FastStart TaqMan Probe Master (Roche Diagnostics, Basel, Switzerland) were used in each PCR. A fluorescence signal was captured during each of the 45 cycles (denaturizing for 10 s at 95 °C, annealing for 20 s at 60 °C, and extension for 1 s at 72 °C). Water was used as a negative control. Relative quantification was calculated using the comparative threshold cycle (CT), which is inversely related to the abundance of mRNA transcripts in the initial sample. The mean CT of duplicate measurements was used to calculate ΔCT as the difference in CT for target and reference. The relative quantity of product was expressed as fold induction of the target gene compared with the reference gene according to the formula 2^−ΔΔ*C*T^, where ΔΔ*C*T represents Δ*C*T values normalized with the mean Δ*C*T of control samples.

### 4.3. Oxidative Stress Gene Expression PCR Array in Cultured Cells

HepG2 cells were seeded (8 × 10^5^ cells per well) in 12-well plates and incubated alone or treated with H_2_O_2_ in the presence of CeO2NPs (10 μg/mL) or vehicle for 24 h to assess changes in oxidative stress pathways. Total RNA was extracted using TRIZOL as described above. To remove residual DNA, RNA preparations were treated with RNase-Free DNAse set (Qiagen, Hilden, Germany). First-strand cDNA was synthesized from 1 μg total RNA using an RT2 first-strand kit (Qiagen, Hilden, Germany), and PCR arrays were performed according to the manufacturer’s protocols (SABiosciences, Frederick, MD, USA). Real-time PCR array was performed using the Human Oxidative Stress RT2 Profiler™ PCR array, (SABiosciences, Frederick, MD, USA) according to the manufacturer’s protocol. This PCR array combines the quantitative performance of SYBR Green-based real-time PCR with the multiple gene profiling capabilities of microarray to profile the expression of 84 key genes involved in oxidative stress. PCR array plates were processed in a Light Cycler 480 (Roche Diagnostics, Basel, Switzerland) using automated baseline and threshold cycle detection. Gene expression was normalized to internal controls to determine the fold change in gene expression between test and control samples. The relative quantity of product was expressed as fold-induction of the target gene compared with the reference gene according to the formula 2^−ΔΔ*C*T^. Data were interpreted using the SABiosciences’ web-based PCR array data analysis tool. Statistical significance was obtained after performing a Student’s t-test analysis compared to control samples (Available online: http://pcrdataanalysis.sabiosciences.com/pcr/ arrayanalysis.php).

### 4.4. Statistical Analysis

Quantitative data were analyzed using GraphPad Prism 5 (GraphPad Software Inc., San Diego, CA, USA), and statistical analysis of the results was performed using one-way analysis of variance (ANOVA) with the Newman-Keuls post hoc test and the Kruskal-Wallis test with the Dunn post hoc test when appropriate. Results are expressed as mean ± SE and considered significant at a p level less than 0.05. The study was performed according to the criteria of the Investigation and Ethics Committee of the Hospital Clínic Universitari of Barcelona.

### 4.5. Phosphoproteomic Analysis

Large-scale phosphoproteomics was used to gain further insight on the kinase signaling pathways mainly affected by the CeO_2_NPs treatment. Thousands of phosphorylation sites were simultaneously quantified to estimate changes in kinase activity induced by CeO_2_NPs in HepG2 cells under oxidation-induced conditions. HepG2 cells were cultured as described above. Thereafter, the cells were switched to serum free medium for 24 h. Then medium was replaced and the cells were exposed to 1.5 mM H_2_O_2_ (Sigma-Aldrich, St. Louis, MO, USA), 1.5 mM H_2_O_2_ and 10 µg/mL CeO_2_NPs or vehicle (TMAOH, 0.17 mM) for 1 h. Confluent cells were washed three times with cold PBS supplemented with 1 mM Na_3_VO_4_ and 1 mM NaF and lysed in urea buffer (8 M urea in 20 mM HEPES, pH: 8.0, supplemented with 1 mM Na_3_VO_4_, 1 mM NaF, 1 mM Na4P2O7 and 1 mM β-glycerophophate). Five independent biological replicates were collected for each condition and kept at −80 °C. Cell lysates were further homogenized by sonication (30 cycles of 30 s on 30 s off; Diagenode Bioruptor^®^ Plus, Liege, Belgium) and insoluble material was removed by centrifugation. Protein was quantified using BCA (Thermo Fisher Scientific, Waltham, MA, USA). For tryptic digestion, protein extracts (500 μg) were subjected to cysteine alkylation using sequential incubation with 10 mM dithiothreitol (DDT) and 16.6 mM iodoacetamide (IAM) for 1 h and 30 min respectively at 25 °C and agitation. The urea concentration was then reduced to 2 M by the addition of 20 mM HEPES (pH: 8.0). Then, 100μL of equilibrated trypsin beads ((50% slurry of TLCK-trypsin (Thermo-Fisher Scientific; Cat. #20230, Waltham, MA, USA) were added, and samples were incubated overnight at 37 °C. Trypsin beads were equilibrated by 3 washes with 20 mM HEPES (pH: 8.0). Trypsin beads were removed by centrifugation and the resulting peptide solutions were desalted with C-18-Oasis cartridges as indicated by the manufacturer. Briefly, oasis cartridges were conditioned with 1 mL acetonitrile (ACN) and equilibrated with 2.5 mL of wash solution (0.1% trifluoroacetic acid (TFA) and 2% ACN). Peptides were loaded in the cartridges and washed with 1 mL of wash solution. Finally, peptides were eluted with 0.5 mL of glycolic acid buffer 1 (1 M glycolic acid, 5% TFA, 50 % ACN). Enrichment of phosphorylated peptides was performed with TiO_2_. The eluents were normalized to 1 mL with glycolic acid buffer 2 (1 M glycolic acid, 5% TFA, 80 % ACN) and incubated with 50 µl of TiO_2_ buffer (a 50% slurry in 1% TFA) for 5 min at room temperature. TiO_2_ beads were packed by centrifugation in empty spin columns (Gygen Corporation; Cat. TT2EMT) previously equilibrated using glycolic acid, 50% ACN and ammonium bicarbonate buffer (20 mM NH_4_HCO_3_ (pH: 6.8) in 50% ACN). For phosphopeptide elution, beads were incubated for 1 min at room temperature with 50 µL of 5% NH_4_OH in 50% ACN and centrifuged. This step was repeated three times. Finally, samples were snap frozen, dried in a SpeedVac, and the pellets were stored at −80 °C.

Phosphopetide pellets were resuspended in 9 µL of reconstitution buffer (20 fmol/µL enolase in 3% ACN, 0.1% TFA) and 5.0 µL were loaded onto an LC-MS/MS system consisting of a Dionex UltiMate 3000 RSLC directly coupled to an Orbitrap Q-Exactive Plus mass spectrometer (Thermo Fisher Scientific, Waltham, MA, USA). Phosphopeptides were loaded in a μ-pre-column (Acclaim™ PepMap™ 100 C18 LC; Cat 160454, Thermo Fisher Scientific, Waltham, MA, USA) and separated in an analytical column (Acclaim™ PepMap™ 100 C18 LC; Cat. 164569, Thermo Fisher Scientific, Waltham, MA, USA) using a gradient that runs from 3% to 23% B over 120 min. The UPLC system delivered a flow of 2 µL/min (loading) and 300 nL/min (gradient elution). Solvent A consists of 3% ACN: 0.1% FA and solvent B consists of 100% ACN; 0.1% FA. The Q-Exactive Plus acquired full scan survey spectra (m/z 375–1500) with a 70,000 FWHM resolution followed by data-dependent acquisition in which the 20 most intense ions were selected for HCD (higher energy collisional dissociation) and MS/MS scanning (200–2000 m/z) with a resolution of 17,500 FWHM.

Peptide identification was performed by matching of the MS/MS data to the SwissProt database, restricted to human entries with the Mascot search engine [48]. Phosphopeptides with a mascot expectancy of < 0.005 (~2% false discovery rate) were included in a database of sites quantifiable by MS. Pascal software [49,50,51] was then used to obtain peak areas of extracted ions chromatograms of phosphorylated peptides in this database across all the samples compared. The significance of the differences in the log2-transformed data across samples was assessed using the Student’s t-test. Inference of kinase activities from the phosphoproteomic data was performed using kinase substrate enrichment analysis (KSEA), as described previously [18].

## 5. Conclusions

Our study shows that CeO_2_NPs directly protect human-derived hepatocytes from oxidative damage, reducing ROS generation and inflammatory gene expression, thus opening new avenues to use CeO_2_NPs in human liver diseases. Additionally, specific cell kinase driven signaling pathways downregulated by CeO_2_NPs treatment in cells under oxidative stress have been identified for the first time. Further experiments with appropriate inhibitors are needed to demonstrate a direct cause-effect relationship between CeO_2_NPs and the modulation of mTOR, ERK and PKACA pathways. Moreover, additional in vivo validations are necessary to determine the potential clinical applications of these findings.

## Figures and Tables

**Table 1:** Dynamic light scattering (DLS) and Zeta potential (Z-Pot) values of CeO_2_NPs purified and resuspended in TMAOH 10 mM and after exposure to DMEM + 10% FCS (CCM). A decrease of the Z-Potential towards the value of the FCS (Z-Pot = −10 mV) and an increase of the hydrodynamic diameter due to the absorption of proteins can be observed. The increase of DLS and maintenance of size distribution observed by TEM images after 2 days in CCM is an indication of the stability of the NPs and the protein corona formation in the CCM.

CeO_2_NPs (TEM = 4–5 nm)		
Table Header	DLS (Z-average, nm)	Z-Pot (mV)
NPs after purification (in TMAOH 10 mM)	33.0	−47.0
0 d in cCCM	70	−35.4
1 d in cCCM	70	−36.4
2 d in cCCM	72.9	−35.0
15 d in cCCM	79	−16.9
30 d in cCCM	100.2	−9.6

**Table 2:** Messenger expression of genes involved in oxidative stress and antioxidant defense in HepG2 cells exposed to H_2_O_2_.

Genes	H_2_O_2_ (*n* = 5)	H_2_O_2_ + CeO_2_NPs (*n* = 6)
*Antioxidants*		
*CAT*	−2.06 **	−2.67 ***
*GPX7*	−2.01 *	−2.25 ***
*LPO*	3.54	1.72
*MPO*	5.32 *	1.54 *†
*TTN*	3.13	1.78
*PTGS1*	3.69 *	1.07 †
*SOD3*	−3.28 **	−6.30 ***
*SRNX1*	3.99 **	2.91 **
*TXNRD1*	3.07 *	2.16
*Genes involved in ROS metabolism*		
*NCF1*	2.59 *	1.38 *
*NCF2*	1.11	−1.89 †
*UCP2*	−2.30 *	−4.04 ***
*EPHX2*	−3.51 ***	−4.36 ***
*Oxidative stress responsive genes*		
*DCHR24*	−2.66 **	−3.36 ***
*DUSP1*	8.30 *	9.39 *
*FOXM1*	−3.20 **	−3.44 ***
*GCLC*	3.29 ***	3.42 ***
*GCLM*	3.86 **	2.30 *
*HMOX1*	2.68	3.15
*HSPA1A*	5.28 *	2.42
*MBL2*	−3.32 *	−2.6 **
*OXR1*	−2.08 ***	−2.64 ***
*SCARA3*	−5.11 **	−7.08 **
*SEPP1*	−4.19 ***	−4.41 ***
*Oxygen transporters*		
*MB*	−5.88 *	−5.84 **

CAT: catalase; GPX7: glutathione peroxidase 7; LPO: lactoperoxidase; MPO: myeloperoxidase; TTN: titin; PTGS1: prostaglandin-endoperoxide synthase 1 (prostaglandin G/H synthase and cyclooxygenase); SOD3: superoxide dismutase 3; SRXN1: sulfiredoxin 1 homolog; TXNRD1: thioredoxin reductase 1; NCF1: neutrophil cytosolic factor 1; NCF2: neutrophil cytosolic factor 2; UCP2: uncoupling protein 2 (mitochondrial, proton carrier); EPHX2: epoxide hydrolase 2, cytoplasmic; DCHR24: 24-dehydrocholesterol reductase; DUSP1: dual specificity phosphatase 1; FOXM1: forkhead box M1; GCLC: glutamate-cysteine ligase, catalytic subunit; GCLM: glutamate cysteine ligase, modifier subunit; HMOX1: heme oxygenase (decycling) 1; HSPA1A: heat shock 70kDa protein 1A; MBL2: mannose-binding lectin (protein C) 2, soluble; OXR1: oxidation resistance 1; SCARA3: scavenger receptor class A, member 3; SEPP1: selenoprotein P, plasma, 1; MB: myoglobin. * *p* < 0.05, ** *p* < 0.01, *** *p* < 0.001 vs. control; † *p* < 0.05 vs. H_2_O_2_ + CeO_2_NPs (unpaired Student’s *t*-test).

**Table 3:** Phosphopeptides with significantly decreased phosphorylation with a Log2 fold ≤ −2 after treatment with CeO_2_NPs in HepG2 cells exposed to H_2_O_2_.

Protein	Phosphopeptide	H_2_O_2_ vs Control. Fold	H_2_O_2_ + NPs vs H_2_O_2_ Fold
AT-rich interactive domain-containing protein 1A	ARID1A seq: 1182–1202 + Phospho (ST)	3.0099 *	−2.727 *
Charged multivesicular body protein 2b	CHMP2B pS199	9.963 *	−9.964 *
C-Jun-amino-terminal kinase-interacting protein 4	SPAG9 seq: 223–241 + Phospho (ST)	10.117 *	−10.11 *
Deoxynucleotidyltransferase terminal-interacting protein 2	DNTTIP2 pS141	10.135 *	−10.13 *
DNA replication licensing factor MCM3	MCM3 seq: 696–724 + Gln- > pyro-Glu (N-term Q); Phospho (ST)	8.390 *	−8.391 *
DNA-dependent protein kinase catalytic subunit	PRKDC seq: 3197–3232 + Phospho (ST)	8.6913 *	−8.692 *
Double-stranded RNA-binding protein Staufen homolog 1	STAU1 pS390	8.9250 *	−8.926 *
E3 ubiquitin-protein ligase UBR5	UBR5 seq: 636–654 + Phospho (ST)	10.128 *	−10.12 *
Eukaryotic translation initiation factor 4E-binding protein 1	EIF4EBP1 pS65 pT70	10.841 *	−10.84 *
Kanadaptin	SLC4A1AP pS466	9.2633 *	−9.264 *
Kanadaptin	SLC4A1AP seq: 324–362 + Oxidation (M); Phospho (ST)	9.164 *	−9.165 *
MKL/myocardin-like protein 2	MKL2 seq: 535–562 + Phospho (ST)	9.971 *	−9.973 *
Pericentriolar material 1 protein	PCM1 seq: 1923–1972 + Phospho (ST); Phospho (Y)	10.677 *	−10.67 *
Prolyl 3-hydroxylase OGFOD1	OGFOD1 seq: 381–427 + Phospho (ST)	9.012 *	−9.013 *
Protein PRRC2A	PRRC2A seq: 1103–1128 + Gln- > pyro-Glu (N-term Q); Phospho (ST)	9.2358 *	−9.236 *
R3H domain-containing protein 1	R3HDM1 seq: 295–314 + Phospho (ST)	8.592 *	−8.593 *
Ras-responsive element-binding protein 1	RREB1 seq: 1636–1665 + Phospho (ST)	9.296 *	−9.297 *
Stress-70 protein. mitochondrial	HSPA9 pM370 pM389	11.353 *	−11.35 *
Telomeric repeat-binding factor 2	TERF2 seq: 404–447 + Phospho (ST)	8.8833 *	−8.884 *
Transcriptional coactivator YAP1	YAP1 seq: 162–181 + Gln- > pyro-Glu (N-term Q); Phospho (ST)	3.3580 *	−5.372 *
Apoptotic chromatin condensation inducer in the nucleus	ACIN1 seq: 463–506 + 3 Phospho (ST)	5.9139 *	−11.89 **
Paxillin	PXN seq: 298–317 + Phospho (ST)	1.8510 *	−2.535 **
Poly(rC)-binding protein 1	PCBP1 pM186 pS190	1.9716 *	−2.646 **
Nascent polypeptide-associated complex subunit alpha	NACA pS2029	1.284 *	−3.119 **
RNA-binding protein 25	RBM25 pS703	11.804 *	−4.246 **
Phosphoribosyl pyrophosphate synthase-associated protein 1	PRPSAP1 seq: 193–220 + Oxidation (M); Phospho (ST)	12.440 **	−4.758 *
Serine/arginine repetitive matrix protein 2	SRRM2 seq: 2275–2301 + Phospho (ST)	9.8054 **	−9.80 *
Cell division cycle protein 23 homolog	CDC23 pT562	8.7452 **	−8.746 **
Centrosomal protein of 131 kDa	CEP131 seq: 45–56 + Phospho (ST)	9.3197 **	−9.320 **
Deoxynucleotidyltransferase terminal-interacting protein 2	DNTTIP2 pS381	9.412 **	−9.41 **
Nuclear receptor coactivator 2	NCOA2 pS771	2.5932 **	−2.630 **
Oxysterol-binding protein 1	OSBP seq: 377–395 + 2 Phospho (ST)	2.8700 **	−2.558 **
RNA-binding motif protein. X chromosome	RBMX pS208	9.8237 **	−9.824 **
Transcription factor Sp5	SP5 seq: 43–68 + Phospho (ST)	4.5004 **	−10.69 **
Uncharacterized protein C6orf106	C6orf106 seq: 264–287 + Phospho (ST)	8.2914 **	−2.875 **
Epsin-1	EPN1 seq: 412–445 + Phospho (ST)	13.834 **	−4.391 ***
Pinin	PNN pS66	8.894 ***	−4.875 *
Protein LYRIC	MTDH pS298	8.390 ***	−8.391 **
POU domain. class 2. transcription factor 1	POU2F1 seq: 273–293 + Phospho (ST)	8.691 ***	−8.69 ***

Statistical significance was assessed using the t-test. *. *p* < 0.05; **. *p* < 0.01; ***. *p* < 0.001.

## Abbreviations

2’,7’-DCF-DA	2′,7′-dichlorofluorescin diacetate
CeO_2_NPs	Cerium Oxide Nanoparticles
DDT	Dithiothreitol
DMEM	Dulbecco’s Modified Eagle Medium
FCS	Fetal Calf Serum
HBSS	Hank’s Balanced Salt Solution
HCC	Hepatocellular Carcinoma
HCD	Higher Energy Collisional Dissociation
HR-TEM	High Resolution Transmission Electron Microscopy
KSEA	Kinase-Substrate Enrichment Analysis
LC-MS/MS	Liquid Chromatography Tandem Mass Spectrometry
LPS	Lipopolysaccharide
MS	Mass Spectrometry
MS/MS	Tandem mass spectrometry
NPs	Nanoparticles
PCR	Polymerase Chain Reaction
ROS	Radical Oxygen Species
RT-PCR	Real Time PCR
SOD	Superoxide Dismutase
UPLC	Ultrahigh Pressure Liquid Chromatography
UV-VIS	Ultraviolet-Visible
XRD	X-ray Diffraction
